# Supplementary figures and images for: Distinct Neuromodulatory Effects of Endogenous Orexin and Dynorphin Corelease on Projection-Defined Ventral Tegmental Dopamine Neurons
Source: J Neurosci. 2024 Aug 26;44(39):e0682242024. doi: 10.1523/JNEUROSCI.0682-24.2024 (PMC11426376; doi:10.1523/JNEUROSCI.0682-24.2024)

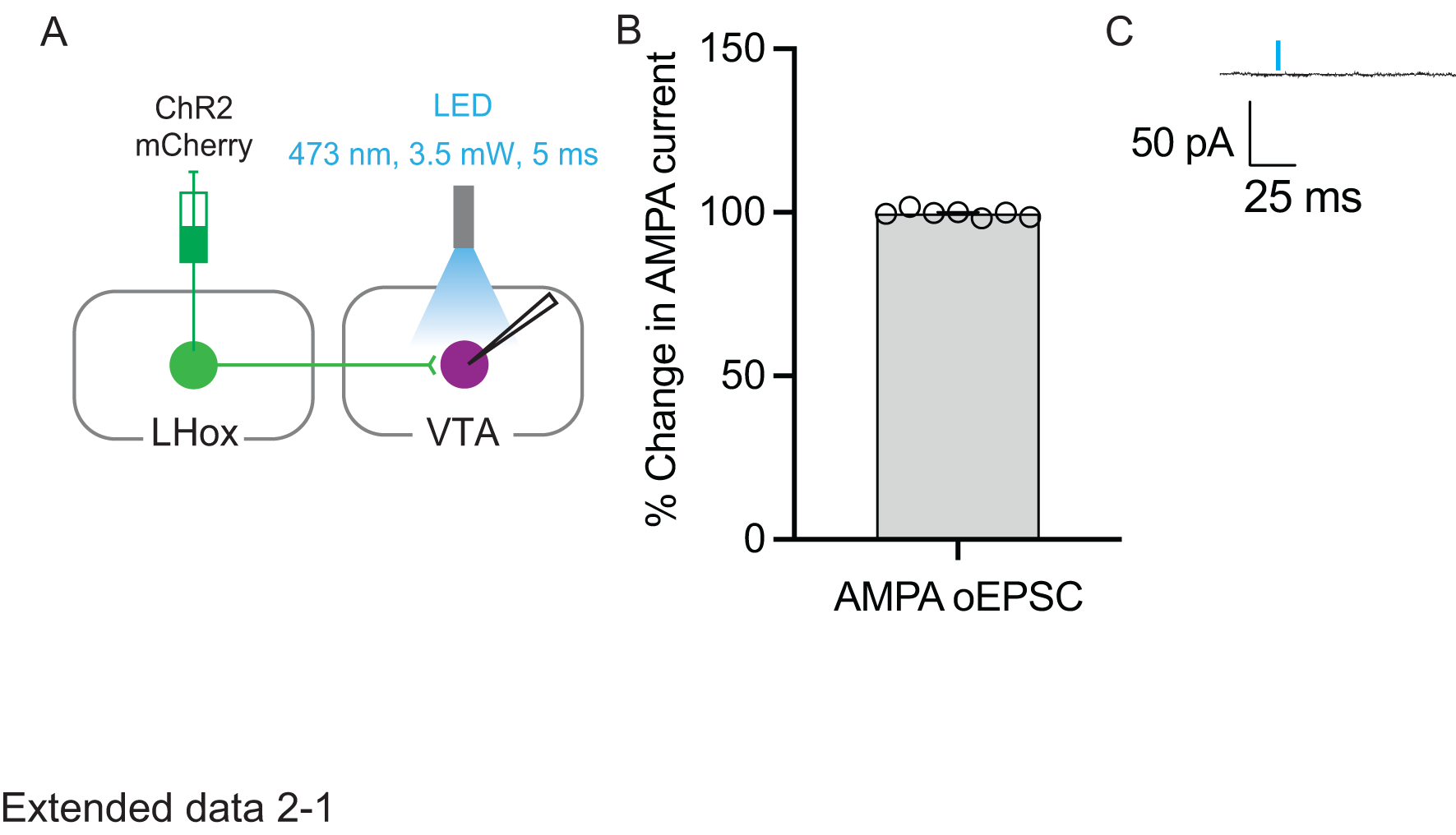

Supplement: Figure 2-1 — Optical stimulation of LHox/dyn inputs does not produce AMPA EPSCs in the VTA. A) Diagram of parameters used to optically stimulate AMPA EPSCs recorded at -70 mV in the presence of picrotoxin in the VTA. B) Percent change in response post optical stimulation compared to pre-stimulation baseline. C) Example sweep from a neuron recorded before and after optical stimulation. Download Figure 2-1, TIF file. [file jneuro-44-e0682242024-s001.tif]

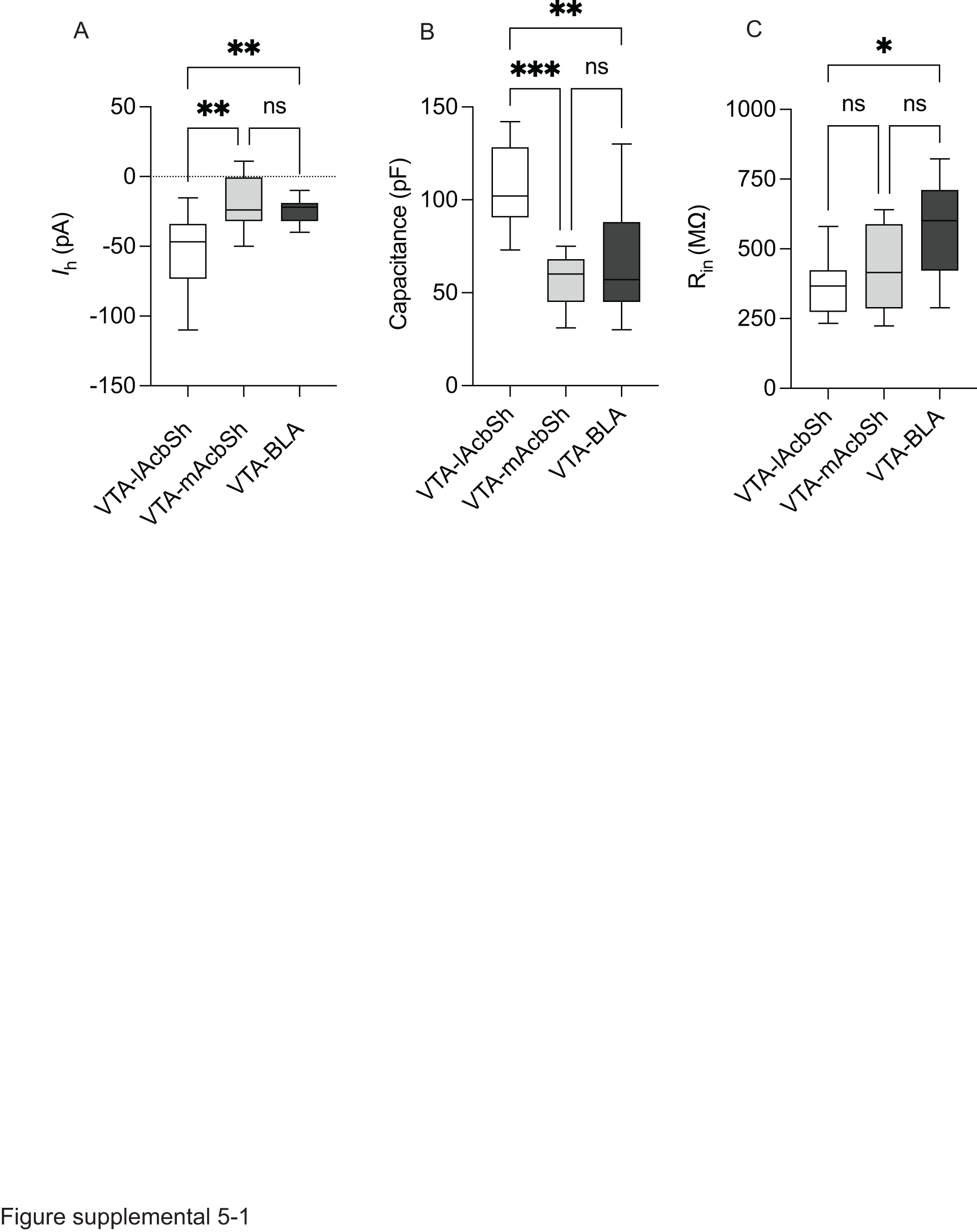

Supplement: Figure 5-1 — lAcbSh-, mAcbSh-, or BLA-projecting VTADA neurons have different intrinsic electrophysiological properties. A) HCN current B) capacitance and C) input resistance of lAcbSh- (open bars), mAcbSh- (shaded bars)- and BLA- (filled bars) projecting VTADA neurons recorded from ChR2 orexincre mice. Download Figure 5-1, TIF file. [file jneuro-44-e0682242024-s002.tif]
